# The Effect of Garlic and Voluntary Exercise on Cardiac Angiogenesis in Diabetes: The Role of MiR-126 and MiR-210

**DOI:** 10.5935/abc.20190002

**Published:** 2019-02

**Authors:** Roya Naderi, Gisou Mohaddes, Mustafa Mohammadi, Alireza Alihemmati, Amirmahdi Khamaneh, Rafighe Ghyasi, Rana Ghaznavi

**Affiliations:** 1 Nephrology and Kidney Transplant Research Center - Urmia University of Medical Sciences, Urmia - Iran; 2 Department of Physiology, faculty of Medicine - Urmia University of Medical Sciences, Urmia - Iran; 3 Neuroscience Research Centre of Tabriz University of Medical Sciences, Tabriz - Iran; 4 Drug Applied Research Center of Tabriz University of Medical Sciences, Tabriz - Iran; 5 Department of Histology and Embryology, Faculty of Medicine, Tabriz University of Medical Sciences, Tabriz - Iran; 6 School of advanced medical sciences - Tabriz University of Medical Sciences, Tabriz - Iran; 7 Sports medicine research center, Neuroscience institute - Tehran University of Medical Sciences, Tehran - Iran

**Keywords:** Rats, Garlic, Allium Sativum, Exercise, Diabetes Mellitus, microRNAs, Angiogenesis Inducing Agents, Neovascularization, Physologic

## Abstract

**Background:**

Diabetes mellitus (DM) is one of the major risk factors for cardiovascular disease, leading to endothelial dysfunction and angiogenesis impairment . MiR-126 and miR-210 support angiogenic response in endothelial cells.

**Objective:**

The present study sought to explore the effect of garlic and voluntary exercise, alone or together, on miR-126 and miR-210 expressions and cardiac angiogenesis in rats with type 1 diabetes.

**Methods:**

Male Wistar rats were divided into five groups (n = 7): Control, Diabetes, Diabetes+Garlic, Diabetes+Exercise, and Diabetes+Garlic+Exercise. Diabetes was induced in the animals by streptozotocin (ip, 50 mg/kg). The rats were then fed raw fresh garlic homogenate (250 mg/kg) or were subjected to voluntary exercise, or to combined garlic and voluntary exercise for 6 weeks. MiR-126 and miR-210 expressions in the myocardium were determined by real time PCR, and the serum lipid profile was measured by enzymatic kits. Angiogenesis was evaluated by immunostaining for PECAM-1/ CD31 in the myocardium.

**Results:**

Diabetes reduced both cardiac miR-126 expression and angiogenesis (p < 0.05). On the other hand, there was a miR-210 expression increase in the myocardium of diabetic animals (p < 0.001). However, those effects reversed either with garlic or voluntary exercise (p < 0.01). Moreover, treating diabetic rats with garlic and voluntary exercise combined had an additional effect on the expressions of miR-126 and miR-210 (p < 0.001). Furthermore, both voluntary exercise and garlic significantly improved serum lipid profiles (p < 0.001).

**Conclusion:**

The induction of diabetes decreased angiogenesis in the myocardium, whereas our treatment using long-term voluntary exercise and garlic improved myocardial angiogenesis. These changes were possibly owing to the enhancement of myocardial miR-126 and miR-210 expressions.

## Introduction

Diabetes mellitus (DM) is one of the major risk factors for cardiovascular disease, leading to endothelial dysfunction and angiogenesis impairment.^1^ The current trend on research and health care focuses on providing effective therapy with few side effects and low toxicity that can be regularly used to control diabetes complications.^2^

Exercise is a powerful therapeutic strategy to improve overall cardiovascular health.^3^ However, exhaustive exercise may be problematic as it can cause the production of reactive oxygen species (ROS).^4^ Therefore, voluntary exercise, in which the animal has free access to a running wheel, may be a model with more positive effects.^5^ There is evidence that aerobic training can promote cardiac angiogenesis,^6,7^ in which the vascular endothelial growth factor (VEGF) has a critical role.^5^ However, the underlying mechanisms of exercise have yet to be fully elucidated.

One of the most traditional plants in herbal medicine is *Allium sativum L*, which has been reported to have beneficial health effects. It is used as a therapeutic agent in various disorders such as cancer, cardiovascular disease, and diabetes through different mechanisms, including inhibition or stimulation of angiogenesis.^2,8,9^ Considering the effects of garlic in protecting against cardiovascular disease, as well as its effects on angiogenesis in different tissues, it is interesting to examine the effects of garlic on both myocardial angiogenesis and its related mechanisms.

MiRs are small non-coding RNAs that function in RNA silencing and the post-transcriptional regulation of gene expression.^10^ MiRs are essential intracellular mediators in many processes such as inflammation, mitochondrial metabolism, apoptosis, and angiogenesis, which can be adjusted through exercise.^11^ Therefore, miRs can be clinically useful in the treatment of several disorders. Moreover, miRs are released in urine and in the bloodstream following tissue injury, which makes them useful biomarkers for early detection, diagnosis, and prognosis of disorders. Recently, these molecules have been found to be involved in cardiovascular diseases.^12^ This includes a high expression of miR-126 in the heart endothelium, as well as its involvement in angiogenesis.^12,13^ Circulating levels of miR-126 are reduced in diabetes,^14,15^ suggesting that its deficiency may impair vascularisation.^16^ Moreover, Fasanaro et al.^17^ reported that hypoxia-driven miR-210 supports angiogenic response in endothelial cells and that its blockade by anti-miR transfection inhibits the formation of capillary-like structures.^17^

Many diabetes complications are well-known to be associated with lipid disorders. Indeed, dyslipidemia impairs numerous organs and is recognized as an important factor of many diabetic complications, including vascular abnormalities.^18^

Therefore, the aim of this study was to investigate the effect of voluntary exercise and garlic treatment alone or in combination on miR-126 and miR-210 expressions, serum lipid profile, as well as their relationship with cardiac angiogenesis in diabetes.

## Methods

### Animals and Experimental Design

The Ethics Committee for Animal Experiments approved the study plan, and all experiments were conducted in accordance with the National Institute of Health’s Guide for the Care and Use of Laboratory Animals. Male Wistar rats (200-250 g) were provided by our university’s colony. All animals were housed in a temperature-controlled facility (21-23°C) maintained on a 12:12-h light-dark cycle, with food and water provided ad libitum.

In this study, thirty-five male rats were divided into five groups (n = 7): Control, Diabetes, Diabetes+Garlic, Diabetes+Exercise, and Diabetes+Garlic+Exercise. Control animals received 0.4 mL of sodium citrate buffer, pH 4.5. Diabetes was induced using a single intraperitoneal dose (50 mg/kg) of Streptozotocin (Sigma, St. Louis, Mo, USA). Blood glucose level was measured 72 hours later using a glucometer (Elegance, Model: no: CT-X10 Germany), and induced diabetes was identified if blood glucose level was > 300 mg/dL (16.67 mmol/L).

In this study, sample size was determined based on our similar previous studies.^8,19^

### Voluntary exercise

Rats in the voluntary exercise groups were housed individually in cages with stainless-steel running wheels (1.00 m circumference, TajhizGostar) and were allowed free access to the wheel 24 h per day for 6 weeks. Running distance was monitored daily. If the running distance was below 2000 m/day, that animal was excluded from the study. Sedentary rats were housed in standard holding cages without running wheels for the same period.

### Preparing Garlic Homogenate

Garlic (Allium sativum) bulbs were purchased from a local market. Cloves were peeled, sliced, ground into a paste and then dissolved in distilled water. The garlic homogenate was freshly prepared each day.

### Sampling

At the end of the 6^th^ week, the rats were deeply anesthetized with pentobarbital sodium (35 mg/kg, i.p.), blood samples were collected from the inferior vena cava to measure lipid profile.

Then the heart *was* quickly *removed* through midsternal thoracotomy and the left ventricle was excised, frozen in liquid nitrogen, and stored at deep freeze (-70°C) for later measurements. The myocardium was used for miR extraction, real-time PCR study and angiogenesis determination.

### MiR Extraction and Real-Time PCR

MiR was extracted from the myocardium using miRCURYTMRNA isolation kit (Exiqon, Vedbaek, Denmark) according to the manufacturer’s protocol.^20,21^ The procedure was performed based on the spin column using a proprietary resin as a matrix to separate RNA from other cell components. RNA content and purity were measured using the Nanodrop 1000 spectrophotometer (Thermo scientific, Wilmington, DE 19810 USA). MiR-126 expression profile was obtained for total RNA extracts using universal a cDNA synthesis kit. Briefly, total RNA containing microRNA was polyadenylated and cDNA was synthesized using a poly(T) primer with a 3’ degenerate anchor and a 5’ universal tag (Exiqon, Vedbaek, Denmark). Each cDNA was used as a template for microRNA quantitative real-time PCR by using the SYBR Green master mix (Exiqon, Vedbaek, Denmark). LNA (Locked Nucleic Acid) forward and reverse primer sets (Exiqon, Vedbaek, Denmark) for microRNA are listed in Table 1. Real-time PCR reactions were performed with a Bio-Rad iQ5 detection System (Bio-Rad, Richmond, CA, USA). The amount of PCR products was normalized with housekeeping rno-miR-191 for miR-126 and miR-210.^37^ We used the 2^-(ΔΔCt)^ method to determine the relative quantitative levels of miR-126 and miR-210. Results were expressed as the fold-difference to the relevant controls.

**Table 1:** Target sequence list for miRs

Gene name	Accession number	Target sequence*
rno-miR-191	MIMAT0000440	CAACGGAAUCCCAAAAGCAGCUG
hsa-miR-126	MIMAT0002957	UCGUACCGUGAGUAAUAAUGC
dme-miR- 210	MIMAT0001233	UUGUGCGUGUGACAGCGGCUA

* Sequences were derived from miRBase (www.mirbase.org).

### Immunostaining for PECAM-1/ CD31

To investigate angiogenesis in the myocardium, transversal sections of the ventricles at their midportion were immediately isolated and fixed in 10% buffered-formalin solution, dehydrated in ascending grades of alcohol and embedded in paraffin. Then, serial 3 µm-thick sections were cut from them and floated onto charged glass slides according to standard histological processing. Tissue pieces were deparaffinised in xylene and dehydrated in a graded series of ethanol. Slides were incubated sequentially in proteinase K and 0.3% hydrogen peroxide to block endogenous peroxidase activity. Sections were overlaid by primary antibody CD31 (Santa Cruz, USA) - an angiogenesis marker - and incubated at +4°C overnight. Afterwards, the sections were washed and incubated with standard avidin-biotin complex (ABC; Santa Cruz) according to the protocol. Then the slides were incubated in DAB (Diamino-benzidine, Santa Cruz) as the chromagen, and counterstained with Mayer's hematoxylin. Finally, the sections were cleared in xylene, mounted with Entellan and analyzed with a light microscope.

### Assessment of immunostaining

To evaluate immunostaining, 3 to 5 sections of 1 mm^2^ were randomly selected at a magnification of 400×, depending on the size of the sample section. Both staining intensity and number of positive cells were evaluated semi-quantitatively. Intensity scoring for CD31 staining was obtained within each area at a 400× magnification. Each endothelial cell cluster of immunoreactivity expressing CD31 and forming lumen or vessels was counted as individual microvessels. Vascular structures positive for CD31 were counted for 5 to 6 slides per animal and 10 fields per slide.

To assess immunostaining, we used the granulation tissue as a positive control, and the intensity of the staining was scored as follows: 0 (<10%); 1 (10% to 25%); 2 (25% to 50%); 3 (50% to 75%) or 4 (75% to 100%).^22^

### Lipid profile measurement

Blood samples were obtained from the inferior vena cava, then centrifuged at 3500 rpm for 10 min at 4°C, and serum was collected. Triglycerides serum level was determined by enzymatic kits (ZiestChem Diagnostic kits, Iran) using glycerol as the standard. Additionally, high-density lipoprotein (HDL) and low-density lipoprotein (LDL) levels were determined based on enzymatic methods by diagnostic kits, (ZiestChem, Iran) using cholesterol as the standard.

### Statistical analysis

All results are expressed as mean ± SEM for seven animals, and analyses were performed using SPSS statistical software version 16. All parameters were tested for normality using the theone-sample Kolmogorov-Smirnov test. Data were statistically analyzed using one-way analysis of variance (ANOVA) followed by Tukey’s test. The significant level was set at p < 0.05.

## Results

### Effects of garlic and voluntary exercise on miR-126 in the myocardium

**Figure 1:**
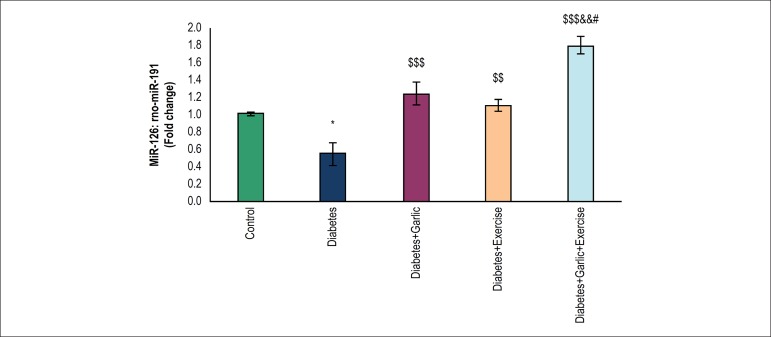
Real-time quantitative PCR analysis of miR-126 in the heart tissue of experimental groups. The values represent means ± S.E.M for 7 animals. *p < 0.05 vs control group, ^$$^p < 0.01 and^$$$^ p < 0.001 vs diabetes group, ^&&^ p < 0.01 vs Diabetes+Exercise group, and ^#^p < 0.05 vs Diabetes+Garlic group.

As shown in Figure 1, myocardial miR-126 expression level was significantly lower (p < 0.05) in rats with diabetes than in the control group. Treatment with garlic (p < 0.001), voluntary exercise (p < 0.01), or both combined increased significantly (p < 0.001) the myocardial miR-126 expression in diabetic rats compared to the diabetes group. Moreover, the Diabetes+Garlic+Exercise group had significantly higher level of miR-126 expression compared to the garlic treatment group (p < 0.05) and the just voluntary exercise group (p < 0.01) in diabetic animals.

### Effects of garlic and voluntary exercise on miR-210 in the myocardium

**Figure 2:**
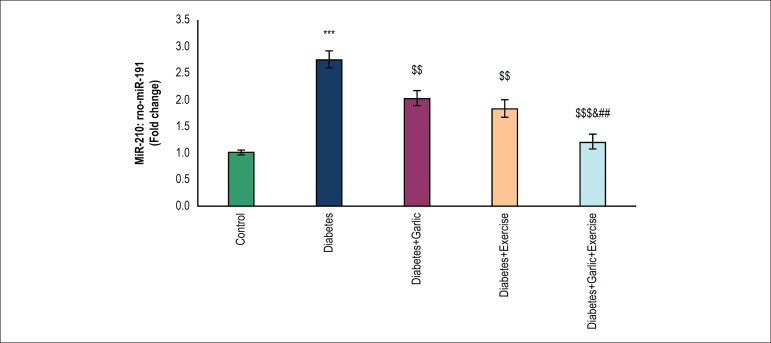
Real-time quantitative PCR analysis of miR-210 in the heart tissue of experimental groups. The values represent means ± S.E.M for 7 animals. ***p < 0.001 vs control group, ^$$^p < 0.01 and ^$$$^p < 0.001 vs diabetes group, ^&^p < 0.05 vs Diabetes + Exercise group, and ^##^p < 0.01 vs Diabetes + Garlic group.

As shown in Figure 2, the expression of miR-210 significantly increased (p < 0.001) in animals with diabetes compared with the control group. Treatment with garlic (p < 0.01), voluntary exercise (p < 0.01), or both combined reduced significantly (p < 0.001) the myocardial miR-210 expression in diabetic rats compared to the diabetes group. The combined Garlic+Voluntary Exercise group significantly lowered miR-210 expression compared to the Diabetes+Exercise (p < 0.05) and Diabetes+Garlic (p < 0.01) groups

### Effect of garlic and voluntary exercise on angiogenesis in the myocardium

**Figure 3:**
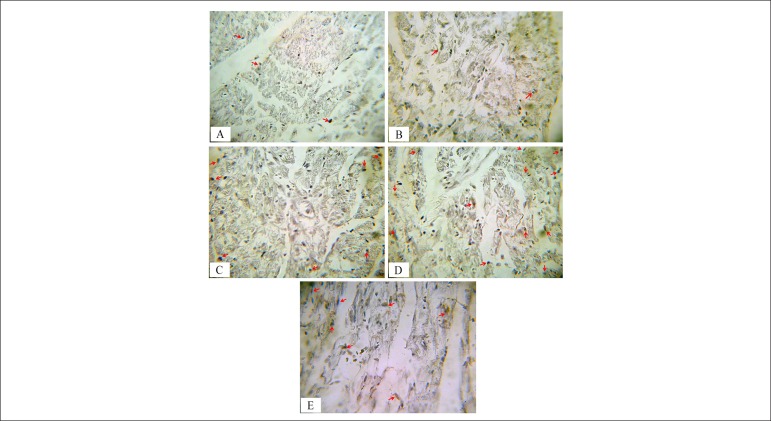
Immunohistochemical detection of CD31 in myocardial vessels of different groups. Brown stained tissues show CD-31 immunostained endothelial cells in: (A) Control; (B) Diabetes; (C) Diabetes+Garlic; (D) Diabetes+Exercise; and (E) Diabetes+Garlic+Exercise. The intensity of immunostaining for CD31 (arrow head) decreased in the diabetes group compared to the control group. Garlic treatment and exercise alone or combined increased angiogenesis in diabetes compared to the diabetes group (Magnification was 400x).

**Figure 4:**
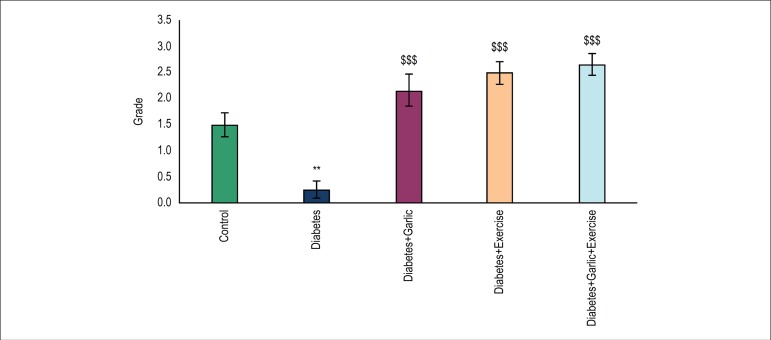
Effects of garlic treatment and voluntary exercise on angiogenesis in different experimental groups. The intensity of the staining was scored as: 0 (<10%); 1 (10-25%); 2 (25-50%); 3 (50-75%); and 4 (75-100%). The values represent means ± S.E.M for 7 animals. **p < 0.01 vs control group and ^$$$^p < 0.001 vs diabetes group.

Immunostaining with CD31 marker was performed for the assessment of angiogenesis in the transversal section of the ventricles at their midportion. Brown stained tissues show CD-31 immunostained endothelial cells. Figure 4 shows the scores for staining intensity, which are as follows: 0 (<10%); 1 (10% to 25%); 2 (25% to 50%); 3 (50% to 75%) or 4 (75% to 100%). As shown in Figures 3 and 4, statistical analysis of our immunohistochemical study revealed that angiogenesis decreased significantly (p < 0.01) in the diabetes group compared to the control group. Six weeks of garlic treatment, voluntary exercise, or a combination thereof in the diabetes groups increased significantly (p < 0.001) the angiogenesis in their left ventricle compared to the diabetes group (Figure 3 and 4). Combined garlic consumption and exercise in diabetic animals induced more angiogenesis compared to garlic alone and exercise alone, though the difference was not significant.

### Effect of garlic and voluntary exercise on serum lipid profile

Lipid profile alterations in different groups are shown in Table 2. The induction of diabetes in the animals increased significantly (p < 0.001) the serum TGs and LDL levels while lowering serum HDL and HDL/LDL compared to the control animals. Voluntary exercise reduced significantly (p < 0.05) the serum triglycerides levels in the diabetes group compared with the control group. Six weeks of garlic treatment alone or with voluntary exercise decreased significantly (p < 0.01) the triglycerides levels in the animals with diabetes. In these, serum LDL levels decreased significantly (p < 0.001) after garlic alone and exercise alone or a combination thereof. However, serum HDL level was significantly increased (p < 0.001) by garlic treatment, voluntary exercise, or a combination thereof in diabetic rats. Furthermore, the HDL:LDL ratio was significantly higher (p < 0.001) in the Diabetes+Garlic, Diabetes+Exercise and Diabetes+Garlic+Exercise groups compared with diabetes group.

**Table 2:** Serum lipid profile in different groups after 6 weeks (Mean ± SEM, n = 7)

Variants	Control	Diabetes	Diabetes+ Garlic	Diabetes+ Exercise	Diabetes+Garlic +Exercise
Triglyceride (mg/dl)	21.3 ± 2.9	87.8 ± 14.3***	42 ± 2.9^$$^	50.1 ± 9.3^$^	44.8 ± 3.7^$$^
LDL(mg/dl)	41 ± 1.69	48.87 ± 1.21***	38.66 ± 0.61^$$$^	39 ± 0.81^$$$^	38.33 ± 0.76^$$$^
HDL(mg/dl)	28.8 ± 1.07	18.25 ± 0.83***	28.16 ± 1.22^$$$^	26.66 ± 1.47^$$$^	27 ± 1.46^$$$^
HDL/LDL	0.7 ± 0.03	0.36 ± 0.01***	0.72 ± 0.03^$$$^	0.67 ± 0.03^$$$^	0.7 ± 0.04^$$$^

*** p < 0.001 vs control group and

$$$ p < 0.001 vs diabetes group. Triglycerides (TG), High-density lipoprotein (HDL), Low-density lipoprotein (LDL)

## Discussion

The present study has shown that the induction of diabetes impaired serum lipid profile, decreased myocardial angiogenesis and miR-126 expression, and increased myocardial expression of miR-210. However, the treatment with garlic alone, voluntary exercise alone or both combined ameliorated these effects in the myocardium of diabetic animals. Interestingly, treating diabetic rats simultaneously with garlic and voluntary exercise had an additional effect on the cardiac expression of miR-126 and miR-210. In line with our study, research has shown that diabetes leads to an impaired function of early endothelial progenitor cells, which results in a reduced capacity of neovascularisation and angiogenesis in the myocardium of diabetic rats.^23^ VEGF, as an inducer of angiogenesis, is a highly specific mitogen for endothelial cells.^24^ It is well-known that the expression of VEGF-A and its receptors decreases in the myocardium of diabetic rats and humans.^25^ However, the actual process of VEGF and angiogenesis reduction in the diabetic heart has not been fully elucidated.

There is a variety of miRs in the heart tissue, and these tiny regulators are recognized as novel targets/drugs in numerous fields, including cardiology.^12^ MiR-126 is known as an endothelial-specific miR that modulates angiogenesis in vivo. Several studies have shown miR-126 to support endothelial homeostasis and angiogenesis,^12,13,15^ which is mediated by SPRED1 and PIK3R2 to promote VEGF signaling.^15^ In addition, miR-126 activates survival kinases such as ERK and Akt by downregulating its targets and promoting the action of VEGF.^26^ Osipova et al reported in their study that urinary miR-126 levels were reduced in the patients with diabetes; however, circulating miR-126 levels in plasma showed no significant difference.^1^

Little information is available about the expression of miR in the myocardium of diabetic rats in response to voluntary exercise. Interestingly, in the present study, we observed that garlic, voluntary exercise and a combination thereof increased the levels of miR-126 expression and angiogenesis in the myocardium. Cardioprotective effects of garlic have been reported in some studies related to improvement of antioxidant activities,^8^ AMPK-mediated AKT/GSK-3β/HIF-1α activation,^27^ and Akt-eNOS signaling pathways.^28^ Moreover, in line with our results, da Silva et al.^6^ showed that aerobic training in healthy rats increased cardiac miR-126 expression, which was possibly related to exercise-induced cardiac angiogenesis.^6^ Furthermore, studies have demonstrated that exercise enhances angiogenesis in the heart both under healthy^29^ and pathological conditions,^5,7^ which highlights the positive effect of physical activity as a non-pharmacological tool in the treatment of cardiovascular disorders. Considering the increased expression of miR-126 following voluntary exercise, cardiac angiogenesis is possibly related to exercise-induced miR-126 expression and VEGF modulation, which upregulates angiogenic pathways such as MAPK and PI3K/Akt/eNOS.^6^

An important hypoxia-induced miR, miR-210 is stimulated following hypoxia and HIF activation.^30^ The elevation of miR-210 gene expression is evidence of hypoxic conditions in the cardiac muscle, in which hypoxia stimulates a number of physiological responses such as angiogenesis through HIF-1α-induced miR-210 expression.^31^ MiR-210 upregulation is a major element of endothelial cell response to hypoxia, which leads to angiogenesis via its target gene Ephrin-A3.^17^ The upregulation of miR-210 and VEGF has been shown to enhance myocardium angiogenesis in acute myocardial infarction in response to Huoxue Anxin Recipe.^32^ Greco et al.^33^ described that, in addition to hypoxia, hyperglycemia is another stimulator that upregulates miR-210 expression, which is observed in diabetes.^33^ Osipova et al.^1^ showed that miR-210 level was upregulated in plasma and urine of type 1 diabetic children,^1^ as well as in cardiomyocytes and endothelial cells in diabetic patients.^33^ In line with these studies, we showed that the induction of diabetes increased myocardial miR-210 level, which was reduced by both garlic, voluntary exercise and a combination of both. Similarly, a recent study demonstrated that plasma miR-210 levels decreased in chronic kidney disease after acute exercise.^34^ On the contrary, some studies have shown that miR-210 was not responsive during acute, exhaustive exercise, sustained aerobic exercise^11^ and swimming^35^ in the heart tissue. Furthermore, both garlic and exercise have been shown to be involved in providing good glycemic control and prevention against long-term diabetic complications.^3,8,19^ Therefore, in the present study, the decrease of miR-210 expression back to normal levels seems to stem from glycemic control. Additionally, garlic extract-mediated angiogenesis probably occurs through the upregulation of the neovasculogenic c-kit protein expression and the activation of the PI3-K/Akt/NF-κB signaling pathways,^36^ which regulates e-NOS activation and NO production.^11^

Hyperglycemia is currently considered to be primarily responsible for the alteration of lipid profile. In general, dyslipidemia is well confirmed in diabetes mellitus; it is known as a criterion for the diagnosis of type I diabetes and potential beta-cell lipotoxin.^37^ It is worth noting that dyslipidemia is related to atherosclerosis and a risk of heart disease.^37^ Dyslipidemia is possibly mediated by the alteration of LXRα expression in the liver and intestine, the activation of nicotinamide adenine dinucleotide phosphate (NADPH) oxidase pathways, and the consequent inhibition of eNOS activity, causing impaired angiogenesis.^15,38^ In addition, dyslipidemia is related to decreased levels of circulating miR-126.^13^ Riedel et al.^39^ showed that exercise in patients with chronic heart failure significantly improved HDL-induced miR-126 expression.^39^ In this study, treatment with garlic and voluntary exercise alone and together ameliorated lipid profile in the serum of diabetic rats, which is in agreement with previous studies.^6,9,40^ Therefore, garlic and exercise have possibly modulated angiogenesis in the myocardium of the diabetic animals by modulating serum lipid profile and the expression of pro-angiogenic miRs. With regard to the limitations of this study, we did not measure other factors involved in angiogenesis. Further studies are necessary to clarify the pathophysiological mechanisms of garlic and voluntary exercise in the treatment of diabetic complications.

## Conclusion

This study showed that garlic and voluntary exercise modulated serum lipid profile and the expression of miR-126, miR-210, thus increasing angiogenesis in myocardium of diabetic rats. These findings suggest that garlic and voluntary exercise alone and combined may hold benefits in the treatment of diabetes.
